# A cross sectional study: latrine coverage and associated factors among rural communities in the District of Bahir Dar Zuria, Ethiopia

**DOI:** 10.1186/1471-2458-13-99

**Published:** 2013-02-04

**Authors:** Worku Awoke, Semahegn Muche

**Affiliations:** 1 College of Medicine and Health Sciences, Bahir Dar University, P.O. Box-693, Bahir Dar, Ethiopia; 2 GAMBY College of Medical Sciences, P.O. Box-209, Bahir Dar, Ethiopia

**Keywords:** Latrine, Latrine coverage facility, Sanitation

## Abstract

**Background:**

Lack of sanitation facilities is a serious health risk and obliges people to practice open defecation, thereby increasing the risk of disease transmission. The aim of this study was to assess latrine coverage and the associated factors among the rural communities in district of Bahir Dar Zuria, Ethiopia.

**Methods:**

A community-based cross-sectional study was conducted on 608 households in district of Bahir Dar Zuria. First, the district was stratified based on the distance from Bahir Dar city. Then, ten kebeles (the smallest administrative units) were selected from the 32 rural kebeles in the district. After the kebeles had been identified, the households were selected by systematic sampling method using existing list of all households as a sampling frame. Intervals (K^th)^) for selecting households were determined by dividing the number of households with the sample size allocated for each kebele. After determining the K^th^ interval, the first household was selected randomly. The next households were identified systematically onwards by adding cumulatively K^th^ intervals to the first selected household .Data were collected by means of a pretested, standardized questionnaire and observation checklist. Data analysis was carried out using SPSS version 16.

**Results:**

Of the 608 households, 355 (58.4%) had pit latrines and only 220 (62.0%) were functional (providing services during data collection). One hundred eighty seven (52.7%) had been constructed two or more years prior to the time of the study and 202 (56.9%) latrines required maintenance. The availability of latrines was twice higher in households with an income of 5000 or more Ethiopian Birr (1USD = 17.5 Ethiopian Birr) per year (adjusted odds ratio [AOR], 1.55; 95% confidence interval [CI], 1.06–2.27) than those who hand an income less than 5000 Birr per year; the availability of latrines was twofold higher in households visited by health professional at least three times a month (AOR, 2.29; 95% CI, 1.33–3.93) than those that received no visits. The latrine coverage was about two times higher in households that were less than 30 minutes walk from a health institution (AOR, 1.57; 95% CI, 1.11–2.22) than households that were over 30 minutes walk. The latrine coverage was lower in households located in distant areas (AOR, 0.53; 95% CI, 0.36-0.77) than in households closer to the city.

**Conclusions:**

Latrine coverage in District of Bahir Dar Zuria was far from the national target of 100%. The availability of latrines was affected by income level, frequency of visits by health workers, walking time from local health institutions, and distance from Bahir Dar. Therefore, it is recommended that the frequency of supportive visits be increased and that special attention be given to households in inaccessible areas.

## Background

Worldwide lack of sanitation is a serious health risk, affecting billions of people around the world, particularly the poor and disadvantaged of people around the world [1,2]. Lack of sanitation facilities compels people to practice open defecation and this increases the risk of transmission of diseases [3]. The disease burden associated with poor water, sanitation, and hygiene is estimated to account for 4.0% of all deaths and 5.7% of the total disease burden in disability-adjusted life year (DALYs) in worldwide, principally through diarrheal diseases, schistosomiasis, trachoma, ascariasis, trichuriasis, and hookworm infection [4]. About 1.8 million people die every year due to diarrheal diseases, and children under the age of 5 years account for 90% of diarrheal deaths. Moreover, 88% of diarrheal diseases are attributed to unsafe water supply, inadequate sanitation, and poor hygiene [5]. The regions with the poorest water supply coverage are sub-Saharan Africa (31%), southern Asia (36%), and Oceania (53%) [3].

In Ethiopia, even though progress was made in reducing child mortality from 123 deaths of under five years of children per 1,000 live births in 2005 [6] to 88 deaths per 1,000 live births in 2011 [7], children in the country still suffer from diarrheal diseases, respiratory problems, and malnutrition. According to Ethiopian demographic and health survey, the two week prevalence of diarrheal diseases was 13% among of children under five years of age [7].To improve sanitation and hygiene throughout Ethiopia, the National Sanitation Strategy establishes the goal of 100% latrine coverage [8]. The construction of sanitation facilities is underway in all parts of the country since the introduction of the Health Extension Program by the Ministry of Health. The present study was conducted to assess the coverage and associated factors regarding the availability of latrines in the Ethiopian rural community of Bahir Dar Zuria, where the Health Extension Program was implemented.

## Methods

### Study design

A community-based cross-sectional quantitative study was conducted from April to May 2012 in the district of Bahir Dar Zuria, Ethiopia. The district has 32 rural *kebeles* (the lowest administrative unit in Ethiopia) and a total population of 194,094. There are nine health centers and 32 health posts in the district. In each kebele, two female health extension workers are assigned to implement the Health Extension Program at the community level.

The sample size was determined by using a single population proportion formula, which considers the proportion of households having latrines as 60.7% [9], with a margin of error of 0.05% at the 95% confidence level. Then multiplying by a design effect of 1.5 and adding a 10% non-response rate, the final sample size was calculated to be 608.

The Bahir Dar Zuria district was stratified based on the distance from the main town, Bahir Dar, as “Near,” “Far,” and “Too Far” (see “Operational Definitions”). Then, a proportional sample size was allocated according to the number of kebeles in each stratum. Finally, ten kebeles were selected from the thirty two rural kebeles in the district. After the study kebeles had been identified, the households were selected by systematic sampling method using the existing list of all households (obtained from registration books of health extension workers in the selected kebeles) as a sampling frame. Intervals (K^th^) for selecting households were determined by dividing the number of households with the sample size allocated for each kebeles. After determining the K^th^ interval, the first household was selected randomly. The next households were identified systematically onwards by adding cumulatively K^th^ intervals to the first selected household.

The study variables were selected after reviewing relevant literatures according to objective of the research and by considering the local context of the study area.The dependent variables was latrine. The independent variables were socio-demographic characteristics, behavioral and environmental factors.

### Data collection and analysis

A structured questionnaire was produced, and an observational checklist was prepared in English before being translated into the local language (Amharic) and then back-translated into English. Sections that showed any discrepancies underwent revision. Ten data collectors and two supervisors were recruited, and a three day training session was given; this mainly dealt with the purpose of the study, handling ethical issues during data collection, and the method of data collection using the prepared questionnaires and observational checklists. Pretesting was carried out on 5% of the sample population prior to data collection at selected health institutions, and corrections were made accordingly. For consistency and completeness, the collected data were checked daily by the supervisors and principal investigators. The collected data were coded, entered, cleaned, and analyzed using the SPSS version 16 software program. Bivariate analysis was conducted to examine the various associations using chi-square test. In addition, crude and adjusted odds ratios (ORs) and 95% CIs were calculated after the chi-square test to test the strength of association and level of significance, respectively. P-Values less than or equal to 5% were considered statistically significant.

### Operational definitions

1. Good latrine: **a pit latrine having superstructure**, **with a door and the possibility of maintaining privacy during defecation**.

2. Fair latrine: **a pit latrine having superstructure**, **without a door but with a leaking roof and sagging walls**.

3. Bad latrine: **a pit latrine without superstructure and lack of privacy during defecation**.

4. Functional latrine: **a latrine that provides services at the time of data collection**.

5. Satisfactory latrine utilization: **households having functional latrines and no observable feces in the compound**, **no observable fresh feces around the squat hole**, **and the footpath to the latrine not being covered by grass**.

6. Near: **households at a distance of 10 kilometers or less from the District Health Office**

7. Far: **households at a distance of 10**–**20 kilometers from the District Health Office**

8. Too far: **households at a distance of over 20 kilometers from the District Health office**

### Ethical considerations

The research topic and methodology were approved by the ethical review committee of Bahir Dar University and GAMBY College of Medical Sciences. Permission to conduct the study was also obtained from the West Gojjam Zonal Health Department and Bahir Dar Zuria District Health Office. Informed oral consent was obtained from the respondents after explaining the purpose of the study. Participants were assured of confidentiality with regard to all information acquired. In addition withdraw from the study during the interview has been guaranteed to all the study participants at any time.

## Results

### Socio-demographic characteristics

A total of 608 households were included in the study. The respondents were either the head of the household or their spouse. Majority (88.7%) of the respondents were married and 479 (78.8%) had a family size of five or more. About 42 (7.8%) fathers and 23 (3.8%) mothers were literate. Among the households, 354 (58.2%) had children attending at primary or junior high school. The majority of the fathers 593 (97.6%) were engaged in farming, and 579 (95.2%) mothers were housewife; 167 (27.5%) households had an income of less than 5000 Ethiopian Birr per year (Table 1).

**Table 1:** **Socio**-**demographic characteristics of respondents**, **Bahir Dar Zuria district**, **May 2012** (**n** = **608**)

**Variable**	**Frequency**	**Percent**
**Family size**		
≥5	479	78.8
<5	129	21.2
**Marital status**		
Married	539	88.7
Divorced	38	6.2
Widowed	31	5.1
**Head of household**		
Father	539	88.7
Mother	69	11.3
**Fathers**’ **occupational status**		
Farmer	593	97.6
Daily Labor	15	2.4
**Children in HHs**		
Not Attending formal education	254	41.8
Attending formal education	354	58.2
**Income**		
<5000 Et. Birr per year	167	27.5
≥5000 Et. Birr per year	441	72.5

### Latrine coverage

Of the households, 355 (58.4%) had latrines. All the available latrine facilities were pit latrines. Of these latrines, 187 (52.7%) were constructed 2 or more years prior to the time of the study. At the time of data collection, 220 (62.0%) latrines were functional. Of the 355 pit latrines, 202 (56.9%) were in need of maintenance, either to the superstructure or floor. Only 97 (27.3%) of latrine had sealed slabs, and only 7 (2%) of the latrines had a cover for the squatting hole.

Of the available latrines, 114 (32.1%) were located at a distance of less than 6 meters from the home. Of the households with latrines, 333 (93.8%) had no any form of hand-washing facilities, and of the 22 households with hand-washing facilities only three used either soap or ash (Table 2).

**Table 2:** **Latrine availability and its condition**, **among the rural community of Bahir Dar Zuria District**, **May 2012**

**Variable**	**Frequency**	**Percent**
**Having latrine **(**n** = **608**)		
Yes	355	58.4
No	253	41.6
**Type of latrine **(**n** = **355**)		
Pit latrine	355	100.0
Others	0	0
**Availability of latrine construction materials **(**n** = **608**)		
Available	551	90.6
Unavailable	57	9.4
**Functional latrine **(**n** = **355**)		
Yes	135	38.0
No	220	62.0
**Latrine squat covered** (**n** = **355**)		
Yes	7	2.0
No	348	98.0
**Latrine year of construction** (**n** = **355**)		
< 2 year	168	47.3
≥2 year	187	52.7
**Latrine condition **(**n** = **355**)		
Needs maintenance	202	56.9
Not need maintenance	153	43.1
**Part of latrine which needs maintenance** (**n** = **202**)		
Super structure	53	26.2
Slab	20	9.9
Roof	113	55.9
Dug	16	7.9
**Availability of Hand washing facility** (**n** = **355**)		
Yes	22	6.2
No	333	93.8
**Detergent used for Hand washing** (**n** = **22**)		
Nothing	18	81.8
Soap	3	13.6
Ash	1	4.6
**Distance of Latrine location from home** (**n** = **355**)		
<6 meters	114	32.1
6-10 meters	102	28.7
>10 meters	139	39.2
**Main source of information for latrine construction **(**n** = **355**)		
Health professionals	180	53.8
Through sanitation campaign	107	31.9
Family members	26	7.7
Mass media	15	4.5
Neighborhoods	7	2.1

In this study, the respondents were asked where they had obtained information about the importance of latrines. Of the respondents who had latrines, 191 (53.8%) explained that they had been advised by health workers to construct latrines, and 113 (31.8%) responded that they had been advised by local administrators as part of the local sanitation campaign Table 2).

### Socioeconomic and environmental determinants of latrine availability

Bivariate analysis was carried out to examine the associated factors for latrine availability at the household level. Many variables were explored to test association of latrine availability and there was no statistically significant association (P-value ≤0.05). Those variables which showed significant association were the household income, availability of latrine construction material, frequency of supportive supervision and walking time from the local health post to the household for supervision and follow-up (P-value ≤0.05).

Adjustment of variables using logistic regression was carried out to predict latrine availability variables that were associated with latrine coverage during the crude analysis. The availability of latrines was about 2-fold higher in households that had an income of 5000 or more birr per year (adjusted OR, 1.55; 95% CI, 1.06 –2.27) than in households with less than 5000 birr per year. The availability of latrines was also two fold higher in households who were visited at least three in a month by health professionals (adjusted OR, 2.29; 95% CI, 1.33 –3.93) than those who received no visits. The latrine coverage was about twice higher in households that were located less than 30 minutes walk time from the local health post (adjusted OR, 1.57; 95% CI, 1.11–2.22) than in households located at a distance of over 30 minutes walk. The latrine coverage was lower in relatively inaccessible areas (adjusted OR, 0.53; 95% CI, 0.36–0.77) than in those closer to the city. Even though the availability of construction materials in the household showed a statistically significant association in the bivariate analysis, this association disappeared in the multivariate analysis (Table 3).

**Table 3:** **The main predictors of latrine availability among the rural community of Bahir Dar Zuria District**, **May 2012** (**n** = **608**)

**Variable**	**Availability of latrine**		**Crude OR**	**Adjusted OR**
	**Yes**	**No**	(**95** % **CI**)	(**95**%**CI**)
**Income**				
<5000 Et.birr per year	109 (65.3%)	58 (34.7%)	1	1
≥5000 Et birr per year	246 (55.8%)	195 (44.2%)	1.49 (1.03-2.16)*	1.55 (1.06-2.27)*
**Frequency of supervision**				
1-2 times per month	211(59.1%)	146 (40.9%	1.38 (0.87-2.20)	1.51 (0.92-2.98)
≥3 times per month	76 (51.0%)	73 (49.0%)	1.92 (1.14-3.24)*	2.29 (1.33-3.93)**
Never visited	68 (66.7%)	34 (33.3%)	1	1
**Walking time from home to health institution**				
≥30 minutes	215 (62.5%)	129 (37.5%)	1	1
<30 minutes	140 (53.0%)	124 (47.0%)	1.48 (1.07-2.04)*	1.57 (1.11-2.22)*
**Kebeles accessibility**/**distance**				
Near -average	236 (55.4%)	190 (44.6%)	1	1
Too far	119 (65.4%)	63 (34.6%)	0.66 (0.46-0.94)*	0.53 (0.36-0.77)**

Significant at p < 0.005*, Significant at p < 0.001**.

## Discussion

The findings of this study shows that 41.6% of the households lacked pit latrines. This result is consistent with the findings of the Ethiopia Demographic and Health Survey in 2011, which indicated that about 45% of rural areas lacked latrine facilities [7]. This finding was also comparable with a study finding from another done in the rural area of Ethiopia 67.7% of latrine coverage was reported [10]. According to the annual report of the district health office of Bahir Dar Zuria 43.6% latrine coverage was reported [11]. Even though it needs further investigation, this discrepancy may be problems in documentation and reporting.

At the time of data collection, 220 (62.0%) latrines were functional (giving services); this figure is lower than that reported in a study conducted in rural Zinder in Niger [12] and (86.7%) reported from study done in Hulet Ejju Enessie district of Ethiopia [9]. Among the available pit latrines, 56.9% required maintenance; this figure is higher than the 47.2% found in a study carried out in 2006 at Hulet Ejju Enessie district of Ethiopia [9].

Despite recommendations to build latrine with a minimum of 6 meters distance from the home in order to avoid the associated health risk and inconvenience [13,14], 114 (32.1%) of the available latrines were located less than 6 meters away from the home.

In Ethiopia 13% of under five children had suffered from diarrhea in the two week period [7] and studies pointed out that hand washing with soap reduces the risk of diarrhea by 48% [15].However in this study, 333 (93.8%) latrine facilities had no any form of hand-washing facilities. This result is consistent with the findings from Kersa Woreda, Eastern Ethiopia, which indicated that about 5.1% of households had a habit of hand washing after defecation [16].

The likelihood of having a latrine was 1.5-fold higher with households that had a higher income than those with a lower income. This finding is in line with the results of a study conducted in 1999 in North Gondar, Ethiopia, [17]. The availability of a latrine was also affected by the frequency of supervision and distance of the household from the local health facility and Bahir Dar city. This could be because households located a short walking distance from the local health facility were better informed about the importance of building latrine facilities and its utilization through health-promotion programs and community mobilization, as was pointed out in studies in northern Ghana [18] and Ethiopia [19].

## Conclusions

In this study, even though there was evidence of improvements over previous trends with respect to latrine coverage in the rural communities of Bahir Dar Zuria district, the 100% latrine coverage was not achieved during the time of data collections. Over half of the available latrines required maintenance. Frequent supportive supervision by health professionals, distance from the local health facility, and income level were the factors that affected latrine coverage. Therefore, it is recommended that the frequency of supportive visits be increased and that special attention be given to households in inaccessible areas.

### Limitations

Some of the data like income, sources of information and use of hand washing facilities and detergents were based on interviews response.

## Abbreviations

AOR: Adjusted Odds ratio; HH: Households; OR: Odds ratio; SPSS: Statistical Package for Social Sciences; WHO: World Health Organization

## Competing interests

The authors declare that they have no competing interests.

## Authors’ contributions

**WA** contributed to proposal development, pre-testing the questionnaires, data cleaning, data analysis, and manuscript preparation. **SM** contributed to proposal development, pre-testing the questionnaire, supervising the data collectors and data entry. Both authors have read and approved the final manuscript.

## Pre-publication history

The pre-publication history for this paper can be accessed here:

http://www.biomedcentral.com/1471-2458/13/99/prepub

## References

[B1] WHO/sanitation URL: http://www.who.int/topics/sanitation/en/

[B2] WHO/10 facts on sanitation URL: http://www.who.int/topics/sanitation/en/

[B3] WHO/10 facts on sanitation URL: http://www.who.int/features/factfiles/sanitation/facts/en/index1.html

[B4] Prüss A Kay D Fewtrell L Estimating the Burden of Disease from Water, Sanitation, and Hygiene at a Global Level Environ Health Perspect 2002 110 5 537 542 10.1289/ehp.02110537 12003760 PMC1240845

[B5] World Health Organization WHO | Facts and figures: Water, sanitation and hygiene links to health 2004 URL: http://www.who.int/water_sanitation_health/publications/factsfigures04/en/

[B6] Central Statistical Agency [Ethiopia] and ORC Macro Ethiopia Demographic and Health Survey 2005 2006 Addis Ababa, Ethiopia and Calverton, Maryland, USA: Central Statistical Agency and ORC Macro

[B7] Central Statistical Agency [Ethiopia] and ICF International Ethiopia Demographic and Health Survey 2011 2012 Addis Ababa, Ethiopia and Calverton, Maryland, USA: Central Statistical Agency and ICF International

[B8] Ministry of Health National Hygiene and Sanitation Strategy 2005 Addis Ababa, Ethiopia http://www.wsp.org/sites/wsp.org/files/publications/622200751450_EthiopiaNationalHygieneAndSanitationStrategyAF.pdf

[B9] Anteneh A Kumie A Assessment of the impact of latrine utilization on diarrhoeal diseases in the rural community of Hulet Ejju Enessie Woreda, East Gojjam Zone, Amhara Region Ethiop J Heal Dev 2010 24 2 110 118

[B10] Ross RK King JD Damte M Evaluation of household latrine coverage in Kewot Woreda, Ethiopia, 3 years after implementing interventions to control blinding trachoma Trop Med Int Health 2011 3 4 251 258 Abstract 10.1016/j.inhe.2011.06.007 24038498

[B11] Bahir Dar Zuria District Health Office 2011/2012 Annual report Bahir Dar Unpublished document from the District Health Office

[B12] Diallo M Hopkins D Kane M Household latrine use, maintenance and acceptability in rural Zinder, Niger Int J Environ Health Res 2007 17 6 443 452 10.1080/09603120701633529 18027197

[B13] Federal Democratic Republic of Ethiopia, Ministry of Health Construction Usage and Maintenance of Sanitary Latrine Extension Package 2004 Addis Ababa http://cnhde.ei.columbia.edu/training/documents/Sanitary_Latrine.pdf

[B14] WHO URL www.who.int/entity/water_sanitation_health/hygiene/…/fs3_4.pdf (accessed December 9, 2012)

[B15] Cairncross S Hunt C Boisson S Bostoen K Curtis V Water, sanitation and hygiene for the prevention of diarrhoea Int J Epidemiol 2010 39 1 193 205 10.1093/ije/dyq035 PMC2845874 20348121

[B16] Mengistie B Baraki N Community based assessment on household management of waste and hygiene practices in Kersa Woreda, Eastern Ethiopia Ethiop J Heal Dev 2010 24 2 103 109

[B17] Admassu M Wubshet M Wubshet T Sanitary Survey in Gondar Town Ethiop J Heal Dev 2004 18 1 39 42

[B18] Rodgers AF Ajono LA Gyapong JO Characteristics of latrine promotion participants and non-participants; inspection of latrines; and perceptions of household latrines in Northern Ghana Trop Med Int Health 2007 12 6 772 782 10.1111/j.1365-3156.2007.01848.x 17550475

[B19] O’Loughlin R Fentie G Flannery B Follow-up of a low cost latrine promotion programme in one district of Amhara, Ethiopia Trop Med Int Health 2006 11 9 1406 1415 10.1111/j.1365-3156.2006.01689.x 16930263

